# Uncertainty and Traceability for the CEESI Iowa Natural Gas Facility

**DOI:** 10.6028/jres.109.026

**Published:** 2004-06-01

**Authors:** Aaron Johnson, Tom Kegel

**Affiliations:** National Institute of Standards and Technology, Gaithersburg, MD 20899-, USA; Colorado Experimental Engineering Station Incorporated (CEESI), Nunn, CO 00000, USA

**Keywords:** natural gas facility, CEESI Iowa uncertainty analysis, CEESI traceability to NIST, correlation coefficient, critical flow venturi uncertainty, traceability, turbine meter uncertainty analysis

## Abstract

This paper analyzes the uncertainty of a secondary flow measurement facility that calibrates a significant fraction of United States and foreign flow meters used for custody transfer of natural gas. The facility, owned by the Colorado Experimental Engineering Station Incorporated (CEESI), is located in Iowa. This facility measures flow with nine turbine meter standards, each of which is traceable to the NIST primary flow standard. The flow capacity of this facility ranges from 0.7 actual m^3^/s to 10.7 actual m^3^/s at nominal pressures of 7174 kPa and at ambient temperatures. Over this flow range the relative expanded flow uncertainty varies from 0.28 % to 0.30 % (depending on flow).

## 1. Introduction

In 1999, Colorado Experimental Engineering Station Incorporated (CEESI) constructed a natural gas calibration facility adjacent to a custody transfer station owned by the Northern Border Pipeline Company in Iowa. This article records results of a joint NIST-CEESI project to provide flow measurement traceability of natural gas flows at the CEESI Iowa facility. This facility is used to calibrate more than 70 % of U.S. flow meters used for custody transfer of natural gas. The facility is designed so that a fraction of the gas delivered to the custody transfer station is directed through a high pressure calibration loop. The calibration loop has a maximum flow capacity of 10.7 actual m^3^/s at nominal pressures of 7174 kPa and at ambient temperatures. During a calibration process, a meter under test (MUT) is calibrated using a parallel array of up to nine turbine meter standards (TMS). Each TMS is routinely calibrated *in its place of use* and *at its operating line pressure* using a bank of 21 critical flow venturis (CFVs). Each of the 21 CFVs in the nozzle bank is traceable to the NIST 26 m^3^ pressure-volume-temperature-time (*PVTt*) primary flow standard. This paper provides a detailed uncertainty analysis and traceability for the calibration of a MUT at the CEESI Iowa facility. The results of the analysis indicate that this facility can achieve a relative uncertainty of 0.28 % to 0.3 % (depending on flow) with a coverage factor of *k* = 2.

## 2. Chain of Traceability

**Fig. 1:**
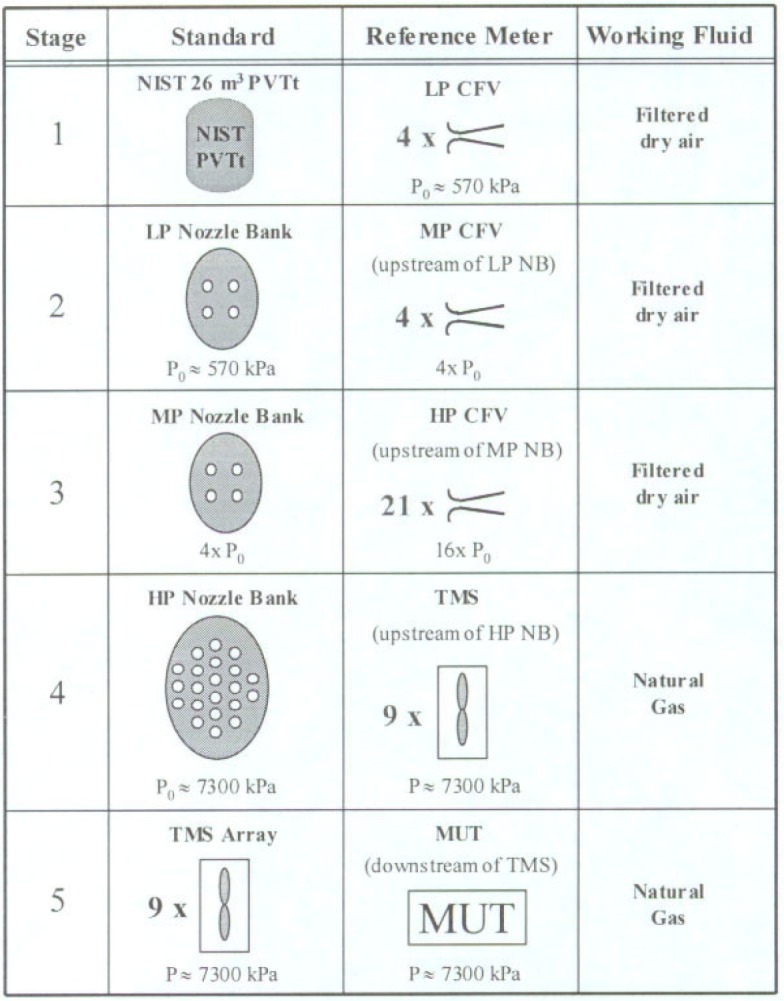
The five stages of traceabillity for a MUT calibrated at CEESI Iowa facility.

Figure 1 shows the five stages of traceability that link the calibration of a MUT at the Iowa facility to the NIST 26 m^3^ *PVTt* flow standard. In Stage 1, the NIST *PVTt* flow standard is used to successively calibrate four low pressure (LP) CFVs. These calibrations are conducted at a nominal pressure of 570 kPa and at ambient temperatures. Stages 2 and 3 consist of a pressure ramp-up process whereby the NIST calibration of the four LP CFVs is transferred first to four medium pressure (MP) CFVs, and then to 21 high pressure (HP) CFVs. All of the CFVs are toroidal shaped and have a nominal throat diameter of 2.54 cm. For the first three stages the working fluid is filtered dry air. In Stage 4, the 21 HP CFVs are mounted in a bank and used to successively calibrate nine TMS. These calibrations are conducted using natural gas as the working fluid. Finally, in Stage 5, the parallel array of nine TMS is used to calibrate a MUT.

The uncertainty of each of the five stages is determined in this document. The analysis accounts for the correlated uncertainties that occur between successive stages. These correlations result from any one of the following: 1) configuring multiple flow meters in parallel (e.g., nozzle bank) that are all traceable to the same calibration standard, 2) using the same transducer to measure nearly identical flow conditions in successive stages, or 3) using two or more transducers (all traceable to the same calibration standard) to measure nearly identical flow conditions within the same stage. Each type of correlation is briefly explained. The analysis includes the governing equations that determine the propagation of uncertainty to avoid ambiguity. We begin with Stage 1 and proceed sequentially to Stage 5.

## 3. Calibration of the LP CFVS

### 3.1 Stage 1 Uncertainty Budget

**Fig. 2:**
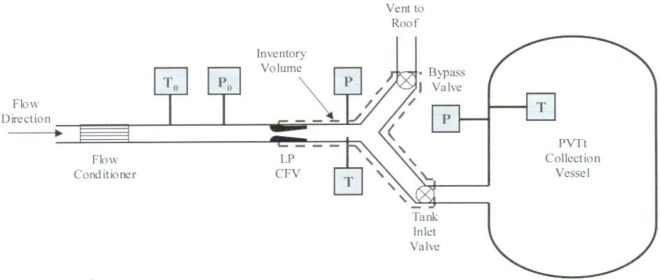
Calibration of LP CFV using the NIST *PVTt* flow standard in 20.4 cm (8 in) pipeline.

The experimental setup for the NIST calibration of the LP CFVs is shown in Fig. 2. The LP CFV is installed in a 20.3 cm diameter pipeline located upstream of the *PVTt* system. The nozzle is calibrated using filtered dry air at nominal pressures of 570 kPa nozzle flow is fully turbulent having a throat Reynolds number of 1.86 × 10^6^.

The main components of the *PVTt* system include the inventory volume, the collection tank, the timing mechanism, the data acquisition system, and the LP CFV. The inventory volume functions to divert the LP CFV flow to either the collection tank or bypass. The collection tank stores the gas, allowing it to equilibrate before determining its mass. The LP CFV plays a dual role, functioning both as the meter being calibrated and as an essential component of the *PVTt* system. In the *PVTt* system the CFV isolates the steady upstream flow from downstream pressure fluctuations that occur during actuation of the bypass and tank inlet valves.

#### Calibration Procedure

The *PVTt* flow standard uses a timed collection technique to determine the LP CFV mass flow. The calibration process begins by establishing steady state flow conditions in the pipeline. The flow emanating from the LP CFV is diverted from the bypass into the nearly evacuated collection tank for a measured time interval. The average gas temperature and pressure in the collection tank are measured before and after the filling process. The density change in the collection tank resulting from the filling process is determined by using an equation of state for dry air [1] along with the measured pressure and temperature. We determine the time-averaged mass flow by the equation

$${\dot{m}}_{PVTt}=\frac{\left(\rho_{T}^{f}-\rho_{T}^{i}\right)V_{T}+\left(\rho_{I}^{f}-\rho_{I}^{i}\right)V_{I}}{\Delta t}$$ (1)

where $\rho_{T}^{f}$ is the final, average gas density in the collection tank; $\rho_{T}^{i}$ is the initial, average gas density in the collection tank; *V*_T_ is the collection tank volume; $\rho_{I}^{f}$ is the inventory volume final average density; $\rho_{I}^{i}$ is the inventory volume initial average density; *V*_I_ is the inventory volume; and Δ*t* is the gas collection time interval. For mass flows between 0.017 kg/s and 1.56 kg/s, the *PVTt* system has an expanded relative uncertainty of 0.13 % (with a coverage factor of *k* = 2). A detailed description of the *PVTt* system and its uncertainty can be found in Refs. [2–4].

The principle of operation for CFVs is largely based on one dimensional isentropic, compressible flow theory [5–7]. This theory is used in CFV metering applications to predict the CFV mass flow. For the steady flow of dry air in Stage 1, the theoretical CFV mass flow is

$${\left({\dot{m}}_{\text{th}}\right)}_{\text{LP}1}={\left[\frac{\pi d^{2}P_{0}C^{*}\sqrt{ℳ}}{4\sqrt{T_{0}R_{u}}}\right]}_{\text{LP}1}$$ (2)

where the subscript “LP1” indicates that all the variables correspond to the low pressure CFV used in Stage 1. In this expression *P*_0_ is the upstream stagnation pressure; *T*_0_ is the upstream stagnation temperature; *d* is the CFV throat diameter; *R*_u_ is the universal gas constant; ℳ is molecular weight of dry air; and C^*^ is the real gas critical flow function for dry air—a function of *P*_0_ and *T*_0_. In this work we calculated C^*^ using a procedure similar to that given by Johnson [8–9].

The key assumption in deriving Eq. (2) is that the Mach number equals unity at the venturi throat. Under this assumption the CFV mass flow is independent of downstream pressure and temperature conditions. This condition is referred to as *choked* flow. Metering applications using CFV flow meters depend on establishing and maintaining choked flow for the duration of the flow measurements.

To achieve choked flow, the pressure ratio across the venturi (i.e., downstream to upstream) must not exceed a minimum threshold called the *critical pressure ratio* (CPR). Researchers have developed analytical methods for estimating the CPR [5–7]. These analytical techniques are suitable when the operating pressure ratio (OPR) is conservatively less than the CPR. In cases where the OPR approaches the predicted CPR, experimental techniques should be used to ensure that choked flow conditions exist. Typically, experimental techniques use *pressure independence test* to verify that pressure changes downstream of the venturui do not influence the upstream pressure and subsequently the mass flow. In Stage 1, choked flow is ensured by maintaining an OPR ratio of 0.14, well below the calculated theoretical CPR of 0.94 for these venturis.^1^

The performance of a CFV can be characterized by two dimensionless variables: the discharge coefficient, *C*_d_, and the Reynolds number, *Re*. The discharge coefficient is a ratio of the actual (i.e., measured) mass flow, ${\dot{m}}_{PVTt}$, to the theoretical mass flow, ${\dot{m}}_{\text{th}}$. We defined *C*_d_ for the LP CFVs in Stage 1

$${\left(C_{d}\right)}_{\text{LP}1}\equiv {\left(\frac{{\dot{m}}_{PVTt}}{{\dot{m}}_{\text{th}}}\right)}_{\text{LP}1}={\left(\frac{4{\dot{m}}_{PVTt}}{\pi P_{0}d^{2}C^{*}}\sqrt{\frac{R_{u}T_{0}}{ℳ}}\right)}_{\text{LP}1}$$ (3)

The isentropic nozzle theory makes no provision for the viscous boundary layer that retards the flow near the venturi wall; therefore, *C*_d_ is less than unity. As the boundary layer thickens at lower Reynolds numbers, *C*_d_ increases. Since the boundary layer thickness is a function of Reynolds number, the discharge coefficient is also a function of Reynolds number. Here we adhere to the following Reynolds number definition

$${\left(Re\right)}_{\text{LP}1}={\left(\frac{4{\dot{m}}_{\text{th}}}{\pi d\;\mu_{0}}\right)}_{\text{LP}1}$$ (4)

where *µ*_0_ is the molecular viscosity evaluated at the upstream stagnation conditions.

#### Expression of Uncertainty for the LP CFV Discharge Coefficient

The uncertainty of the discharge coefficient, *u*(*C*_d_), for the LP CFVs calibrated in Stage 1 is based on the method of propagation of uncertainty [10, 11]. Using this method, *u*(*C*_d_) is given by^2^

$$\begin{array}{l} {\left[\frac{u\left(C_{d}\right)}{C_{d}}\right]}_{\text{LP}1}^{2}={\left[\frac{u\left({\dot{m}}_{PVTt}\right)}{{\dot{m}}_{PVTt}}\right]}^{2}+{\left[\frac{u\left(P_{0}\right)}{P_{0}}\right]}_{\text{LP}1}^{2}+\frac{1}{4}{\left[\frac{u\left(T_{0}\right)}{T_{0}}\right]}_{\text{LP}1}^{2}\\ +4{\left[\frac{u\left(d\right)}{d}\right]}_{\text{LP}1}^{2}+\frac{1}{4}{\left[\frac{u\left(ℳ\right)}{ℳ}\right]}_{\text{LP}1}^{2}+\frac{1}{4}{\left[\frac{u\left(R_{u}\right)}{R_{u}}\right]}^{2}+{\left[\frac{u\left(C^{*}\right)}{C^{*}}\right]}_{\text{LP}1}^{2}. \end{array}$$ (5)

In this expression, the correlations of C^*^ with *P*_0_ and *T*_0_ have been neglected. In a more exact representation the normalized sensitivity coefficients of *P*_0_ and *T*_0_ would include the appropriate pressure and temperature derivatives of C^*^[12]. However, a sensitivity study showed that these dependencies could be omitted with negligible error in both Stages 1 and 2 where the pressure is sufficiently low. On the other hand, we include these dependencies in the uncertainty expressions in Stages 3, 4, and 5 where the pressure is substantially higher. Furthermore, the correlation between *P*_0_ and *T*_0_ (through their common dependence on the specific heat ratio, *γ*, and on the Mach number, *M*, [see Eq. (6)].) affects the uncertainty budget by less than 1 × 10^−6^ and is ignored.

Based on Eq. (5), the Stage 1 uncertainty for the discharge coefficient was 0.067 % with a coverage factor of *k* = 1. Table 1 itemizes the components of this uncertainty. By far, the largest component is the relative standard uncertainty of the *PVTt* primary standard (650 × 10^−6^). This value of uncertainty is documented in the Refs. [2–4]. The remaining uncertainty terms are discussed below.

##### LP CFV Stagnation Pressure and Temperature

The stagnation pressure and temperature are computed using the formulas

$$P_{0}=P{\left[1+\left(\frac{\gamma -1}{2}\right)M^{2}\right]}^{\left(\frac{\gamma }{\gamma -1}\right)},T_{0}=T_{m}\left[\frac{1+\left(\frac{\gamma -1}{2}\right)M^{2}}{1+r\left(\frac{\gamma -1}{2}\right)M^{2}}\right]$$ (6)

where *P* is the static pressure; *T*_m_ is the measured temperature (as indicated by the transducer) before correcting for viscous heating attributed to the flow stagnating against the temperature probe; and *r* = *Pr*^1/3^ is the recovery factor for turbulent flow, which is a function of the Prandtl number, (*Pr*) [5]. These formulas are strictly valid only for ideal gases with a constant heat capacity. However, the low Mach number in Stage 1 (*M* ≈ 0.009) make these formulas reliable even for real gas behavior.

The Stage 1 stagnation pressure is measured using a Paroscientific^3^ Model 740 with a full scale of 1400 kPa. This transducer is calibrated at six month intervals using a piston pressure gauge whose piston area measurement is traceable to the NIST Pressure and Vacuum Group [13]. The relative standard uncertainty components are itemized in Table 2. They consist of the calibration of the pressure transducer (17 × 10^−6^); the manufacturer-specified drift limit (60 × 10^−6^); the calibration fit residuals, hysteresis, and thermal effects (100 × 10^−6^); and the uncertainty attributed to the dynamic pressure (12 × 10^−6^). The combined root-sum-square (RSS) of these components yields a total pressure uncertainty of [*u*(*P*_0_)/*P*_0_] = 118 × 10^−6^.

The temperature is measured using a YSI Model 46000 thermistor in a 3 mm diameter stainless steel sheath. This transducer is calibrated at six month intervals in a uniform temperature bath using results from another thermistor that is directly traceable to the NIST Thermometry Group [13]. The relative standard uncertainty components for temperature are itemized in Table 3. These uncertainty components consist of the uncertainty of the temperature transfer standard (1.2 mK), uniformity of the temperature bath (1 mK), calibration fit residuals (10 mK), manufacturer-specified drift limit (10 mK), heat transfer effects attributed to self heating, stem conduction, and radiation (5 mK), spatial sampling error (50 mK), and the temperature adjustment to account for the moving gas stagnating against the probe (1 mK). The RSS for all of the temperature components yields a total temperature uncertainty of *u* (*T*_0_) = 52 mK.

#### LP CFV Throat Diameter

The venturi throat diameter has two sources of uncertainty: 1) an uncertainty in the nominal throat size, and 2) an uncertainty attributed to expansion (or contraction) of the throat diameter with temperature. The uncertainty attributed to the nominal throat size can be taken equal to zero since the same LP CFVs that are calibrated in Stage 1 are used as transfer standards in Stage 2. Consequently, any uncertainty in the nominal throat diameter present in Stage 1, would completely cancel when the same value for the nominal throat diameter is used in Stage 2.

The uncertainty due to thermal expansion is caused by a change in the throat wall temperature of the LP CFV *between* the time of calibration in Stage 1 and the time of use in Stage 2. As such, thermal effects make no contribution to the uncertainty during the Stage 1 calibration. The uncertainty caused by thermal expansion is only important during the Stage 2 application of the LP CFV. Since neither the nominal throat size nor thermal expansion of the throat make any uncertainty contribution in Stage 1, the total uncertainty is identically zero as indicated in Table 1. This result is general and also applies to the MP CFV and the HP CFV during their respective calibration stages.

#### Molecular Mass

The molecular mass has two sources of uncertainty: 1) an uncertainty attributed to the air moisture level, and 2) an uncertainty resulting from the variation in the composition for dry air. During calibration of the LP CFV the moisture in the air is removed using a desiccant dryer. Based on the manufacturer specifications, the dehumidification process reduces the air moisture content to less than 1 % relative humidity. At a pressure of *P*_0_ = 570 kPa and at room temperature, the amount of substance fraction of water vapor is 4.1 × 10^−5^ resulting in an uncertainty in the molecular mass of 15 × 10^−6^.

Several references list slight variations in the composition of dry air at sea level [14–16]. We estimated that the uncertainty attributed to the variation in composition was 34 × 10^−6^. Assuming a rectangular distribution, the relative standard uncertainty (*k* = 1) is 20 × 10^−6^. Thus, the RSS of the two components yields a total uncertainty of [*u*(ℳ)/ℳ]_air_ = 25 × 10^−6^

#### Universal Gas Constant

The universal gas constant has a value of *R*_u_ = 8314.472 J/(kg · K) with an uncertainty of 2 × 10^−6^ [17].

#### LP Real Gas Critical Flow Function

We determined C^*^ by numerical integration along an adiabat (i.e., line of constant entropy) starting from the stagnation conditions and ending at the *M* = 1 condition. Two sources of uncertainty affect the C^*^ calculation. First, since the integration corresponds to one-dimensional isentropic flow, the calculated value of C^*^ does not account for secondary effects such as the influence that real gas behavior has on the boundary layer development or on the curvature of the sonic line. Second, the uncertainty in C^*^ depends on the uncertainty of the thermodynamic database used to compute the critical flow function.

In some cases gas composition is taken to be the third uncertainty component of C^*^. For example, in Stages 1, 2, and 3 where the working fluid is dry air, the uncertainty in C^*^ due the amount of water vapor in the air is taken to be the third uncertainty component. However, in Stage 4, where the natural gas is the working fluid, the uncertainty analysis for C^*^ includes only the first two components. The uncertainty in C^*^ attributed to gas composition is accounted for in the gas composition uncertainty via the sensitivity coefficients of C^*^ with respect to gas species [see Eq. (20)].

In Stage 1 we made the uncertainty contribution from C^*^ negligibly small. This was accomplished by using the LP CFVs in Stage 2 at the same actual flow conditions (i.e., same working fluid and identical stagnation conditions) that they were calibrated at in Stage 1. In this way, the Stage 1 uncertainty components of C^*^ are identical to those in Stage 2. Moreover, the uncertainty of C^*^ from Stage 1 completely cancels with its corresponding Stage 2 uncertainty, thereby yielding a net zero uncertainty. For simplicity, we implemented this cancellation by setting the uncertainty of the critical flow function equal to zero in both Stages 1 and 2 as shown in Table 1 and later in Table 4. The asterisk next to these uncertainties in the tables indicates our assumption of identical measurement errors.

The flow conditions of the LP CFV between Stages 1 and 2 are kept consistent by controlling the pressure, temperature, and the relative humidity. In Stage 2, the pressure is controlled to within 2 kPa, and a heat exchanger maintains the temperature within 2 K. The relative humidity is maintained below 1 % (at ambient temperatures) by using a desiccant drier. In spite of these small differences between Stage 1 and 2 flow conditions, the uncertainty attributed to the critical flow functions of these stages are assumed to cancel. The uncertainty associated with slight differences between Stage 1 and 2 flow conditions is insignificant relative to the other uncertainty components. These same methods of pressure, temperature, and humidity control are used for the MP CFVs so that the C^*^ uncertainty in Stages 2 and 3 were also assumed to cancel. Finally, the assumption of identical measurement errors due to pressure, temperature, and humidity control does not apply to the HP CFVs since they were calibrated in dry air, but used in natural gas.

## 4. Calibration of the MP CFVS

### 4.1 Stage 2 Uncertainty Budget

**Fig. 3:**
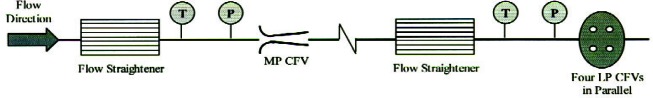
Stage 2 setup for calibrating the MP CFVs using four LP CFVs.

In Stage 2, the four LP CFVs that were calibrated in Stage 1 are used in parallel to calibrate four MP CFVs. Each of the MP CFVs is calibrated separately at nominally the same flow conditions. As shown in Fig. 3, the MP CFV is positioned upstream of the parallel array of LP CFVs. Choked flow conditions are checked using a pressure independence test as explained previously in Stage 1. Since the MP CFV and the four LP CFVs all have the same nominal throat diameter (2.54 cm), the stagnation pressure of the MP CFV will equal approximately four times that of the LP CFVs. The operating conditions for the LP CFVs are controlled so that *P*_0_ and *T*_0_ are nearly equal to their Stage 1 values. Because the working fluid is the same as that in Stage 1 (i.e., dry air), the resulting Reynolds number, and the discharge coefficient will be the same. Slight mismatches in the Reynolds number have a negligible effect on the venturi discharge coefficient. A Reynolds number change of 0.5 % changes the discharge coefficient by less than 3 × 10^−6^ (based on *C*_d_ values calculated using ISO 9300 [12] at *Re* = 1.86 × 10^6^). Since the Stage 1 and 2 Reynolds numbers are expected to agree to better than 0.5 %, the resulting uncertainty is less than 3 × 10^−6^, and is neglected.

#### Calibration Principle

By applying the principle of conservation of mass we determine that the MP CFV mass flow is given by the following expression

$${\dot{m}}_{\text{MP}2}={\sum_{i=1}^{N_{2}}\left[{\dot{m}}_{{\text{th}}_{i}}\right]}_{\text{LP}2}C_{d_{i}}^{LP}+\Delta {\dot{m}}_{2}$$ (7)

where *N*_2_ = 4 is the number of LP CFVs in the array; $C_{d_{i}}^{\text{LP}}$ is the discharge coefficient of the *i*th LP CFV as determined in Stage 1; the Stage 2 theoretical mass flow is determined using an expression analogous to Eq. (2); and $\Delta {\dot{m}}_{2}$ is the rate of mass storage in the connecting volume between the array of LP CFVs and the MP CFV. The mass storage term accounts for density transients in the connecting volume and is commonly called the *line packing* effect. Because $\Delta {\dot{m}}_{2}$ is small relative to the MP CFV mass flow, we set $\Delta {\dot{m}}_{2}$ equal to zero in Eq. (7); however, the uncertainty attributed to the line packing effect is included in the uncertainty budget.

#### Calibration Procedure

The calibration procedure begins by establishing steady-state flow conditions at the desired stagnation conditions. Data is then collected for approximately 120 s. The calibration determines the MP CFV discharge coefficient, $C_{d}^{MP}\equiv {\left(\dot{m}/{\dot{m}}_{\text{th}}\right)}_{\text{MP}2}$, by inverting Eq. (7):

$$C_{d}^{\text{MP}}=\sqrt{\frac{T_{0_{\text{MP}2}}}{T_{0_{\text{LP}2}}}}\left(\frac{P_{0_{\text{LP}2}}C_{\text{LP}2}^{*}}{P_{0_{\text{MP}2}}C_{\text{LP}2}^{*}}\right)\sum_{n=1}^{N_{2}}\left(\frac{d_{{\text{LP}2}_{n}}^{2}}{d_{\text{MP}2}^{2}}\right)C_{d_{n}}^{\text{LP}}$$ (8)

where *d*_LP2_*_n_* is the throat diameter of the *n*th Stage 2 LP CFV after accounting for thermal expansion. The expression for the MP discharge coefficient in Eq. (8) is the instantaneous value that is recorded during the data collection interval. However, the reported values are averaged over the calibration interval.

#### Expression of Uncertainty for the MP CFV Discharge Coefficient

The expression of uncertainty for the discharge coefficient of the MP CFV is determined by applying the law of propagation of uncertainty to Eq. (8); see [10]. The correct application of this procedure requires that the discharge coefficients, $C_{d}^{\text{LP}}$ of the LP CFVs be treated as correlated. The correlation occurs because the characterization of each LP CFV in the array is traceable to the same calibration standard. When this correlation is taken into account, the resulting expression for the relative uncertainty is

$$\begin{array}{l} {\left[\frac{u\left(C_{d}\right)}{C_{d}}\right]}_{\text{MP}}^{2}=\left(\frac{1+r_{\text{LP}}\left(N_{2}-1\right)}{N_{2}}\right){\left[\frac{u\left(C_{d}\right)}{C_{d}}\right]}_{\text{LP}1}^{2}+{\left[\frac{u\left(P_{0}\right)}{P_{0}}\right]}_{\text{MP}2}^{2}\\ +{\left[\frac{u\left(P_{0}\right)}{P_{0}}\right]}_{\text{LP}2}^{2}+\frac{1}{4}{\left[\frac{u\left(T_{0}\right)}{T_{0}}\right]}_{\text{MP}2}^{2}+\frac{1}{4}{\left[\frac{u\left(T_{0}\right)}{T_{0}}\right]}_{\text{MP}2}^{2}+4{\left[\frac{u\left(d\right)}{d}\right]}_{\text{MP}2}^{2}\\ +4{\left[\frac{u\left(d\right)}{d}\right]}_{\text{LP}2}^{2}+{\left[\frac{u\left(C^{*}\right)}{C^{*}}\right]}_{\text{MP}2}^{2}+{\left[\frac{u\left(C^{*}\right)}{C^{*}}\right]}_{\text{LP}2}^{2}+{\left[\frac{\Delta {\dot{m}}_{2}}{{\dot{m}}_{\text{MP}2}}\right]}^{2} \end{array}$$ (9)

where the last term is the uncertainty attributed to line packing effect, *r*_LP_ is the correlation coefficient for the discharge coefficients of the parallel array of LP CFVs, and [*u*(*C*_d_)]_LP1_ is the uncertainty from Stage 1 as given by Eq. (5). In developing Eq. (9) a single representative value of [*u*(*C*_d_)]_LP1_ was used for all four CFVs in the array. This is reasonable since [*u*(*C*_d_)]_LP1_ only varies slightly from venturi to venturi. Theoretically, the value of the correlation coefficient can range from zero (i.e., uncorrelated) to unity (i.e., perfectly correlated). In this work the correlation coefficient was calculated to be *r*_LP_ = 0.95; the method used to calculate this value is discussed here.

#### Correlation Coefficient for the LP CFVs

The correlation between the outputs of different LP CFVs in the nozzle bank is

$$r_{\text{LP}}=\frac{n{\left[\frac{{\left[u\left(C_{d}\right)/C_{d}\right]}_{n}}{{\left[u\left(C_{d}\right)/C_{d}\right]}_{1}}\right]}^{2}-1}{n-1}$$ (10)

where *n* = *N*_2_, [*u*(*C*_d_)/*C*_d_]*_n_* is the uncertainty of the discharge coefficient for the array of *n* LP CFVs, and [*u*(*C*_d_)/*C*_d_]_1_ is the uncertainty for a single LP CFV. The value of [*u*(*C*_d_)/*C*_d_]_1_ was calculated using Eq. (5) and is given in Table 1 of the Stage 1 analysis. The value of [*u*(*C*_d_)/*C*_d_]*_n_* can also be computed using Eq. (5); however, the standard RSS technique for combining the *P*_0_ uncertainty components (Table 2) and the *T*_0_ uncertainty components (Table 3) must be modified. For example, the stagnation temperature uncertainty component is calculated by

$${\left[u\left(T_{0}\right)\right]}_{n}=\sqrt{u_{c}^{2}\left(T_{0}\right)+u_{u}^{2}\left(T_{0}\right)/n}$$ (11)

where *u*_c_(*T*_0_) is the RSS of the correlated sources of uncertainty (i.e., bias components), and *u*_u_(*T*_0_) is the RSS of the uncorrelated sources of uncertainty. For a given measurement, the bias uncertainty components have the same sign and magnitude each time the measurement is repeated. In cases where there is ambiguity as to whether a given component is correlated or uncorrelated, we choose the more conservative approach and define the component to be perfectly correlated. This results in a higher value of the correlation coefficient and consequently a more conservative uncertainty estimate. For example, the bias uncertainty components for the Stage 1 temperature measurements include the temperature transfer standard, uniformity of the temperature bath, calibration fit residuals, heat transfer effects, and the correction for viscous heating. These components are expected to be the same for each of the four LP CFVs calibrated in Stage 1. The remaining components are taken to be uncorrelated between successive calibrations at the same conditions. Dividing *u*_u_(*T*_0_) by *n* in Eq. (11) effectively represents the averaging effect that reduces the uncertainty of the uncorrelated components when multiple venturis are used in parallel. This is equivalent to using the standard deviation of the mean instead of the standard deviation. As would be expected, the bias uncertainties are unaffected by this averaging process. The stagnation pressure uncertainty is computed in an analogous fashion. This method for computing the correlation coefficient is used repeatedly in the successive stages.

Using Eq. (9) the relative standard uncertainty for the discharge coefficient of the MP CFV is calculated to be 749 × 10^−6^ (*k* = 1). An itemized list of the uncertainty components is given in Table 4. As expected, the largest uncertainty contribution derives from the Stage 1 calibration of the four LP CFVs. The value for this uncertainty (655 × 10^−6^) is slightly less than that given in Table 1 of the Stage 1 analysis because of the less than perfect correlation. The remaining uncertainty components are discussed below.

#### Stage 2 LP CFV Stagnation Pressure

In Stage 2 the stagnation pressure of the LP CFVs is measured using a Mensor 15 000 DPG pressure transducer. The transducer is calibrated over a 10:1 pressure range that extends from 345 kPa to 3 450 kPa. This fairly narrow range is chosen to allow some flexibility while simultaneously reducing full scale effects. This pressure transducer is calibrated every 90 days using an Ametek Model RK-300 deadweight tester with a manufacturer specified relative uncertainty of 150 × 10^−6^. This value is taken to be at the 95 % confidence level (*k* = 2).

The transducer’s calibration history spans 2.5 years and includes 79 data points. Instead of using the manufacturer’s quoted value for drift, statistical methods are employed to determine the random effects of the transducer. Based on the control charting methods of Croarkin [18], the random effects are categorized into short term random effects (i.e., random effects occurring during the calibration process) and long term random effects (i.e., random effects occurring between calibration cycles). When the historical calibration data is analyzed using this method, the short term random uncertainty equals 56 × 10^−6^ while the long term random uncertainty is 52 × 10^−6^ both at the *k* = 1 level. 4

The remaining uncertainty components include an uncertainty attributed to the voltage measurement using an Agilent 349070 A data acquisition system (67 × 10^−6^), and the uncertainty attributed to calculating the stagnation pressure from the static pressure measurement (1 × 10^−6^). When all of these uncertainty components are combined using the RSS method, the resulting pressure uncertainty equals 127 × 10^−6^, as shown in Table 5.

#### Stage 2 MP CFV Stagnation Pressure

In Stage 2 the stagnation pressure of the MP CFV is measured using a Mensor 11 900 DPG pressure transducer that has been calibrated over a 10:1 pressure range that extends from 1720 kPa to 17 240 kPa. This Mensor is calibrated in 90 day intervals using a Ruska model 2400 H piston assembly that has a manufacturer specified uncertainty of 100 × 10^−6^ (*k* = 2). This value is assumed to be at the 95 % confidence level. The calibration history of this transducer spans 9.5 years and includes data points. A statistical analysis of the calibration history indicates that the relative uncertainty of the short term random effects and the long term random effects are 180 × 10^−6^ and 172 × 10^−6^ respectively, both at *k* = 1. The uncertainty attributed to the voltage measurement using the Agilent 34 9070 A data acquisition system is 63 × 10^−6^, and the uncertainty attributed to calculating the stagnation pressure from the static pressure measurement is less than 1 × 10^−6^. The RSS of all of the uncertainty components yielded a total uncertainty of 261 × 10^−6^ at *k* = 1. However, since this same transducer is used both in Stage 2 and again in Stage 3, at the same nominal pressure, the bias uncertainty components are perfectly correlated and do not contribute to the uncertainty analysis. Here the RSS of bias uncertainty components total 179 × 10^−6^ and include the calibration transfer standard (50 × 10^−6^), and the long term random effects (172 × 10^−6^). When the bias uncertainty components are omitted, the uncertainty in the stagnation pressure equals 190 × 10^−6^ at *k* = 1 as indicated in Table 6.

#### LP CFV Stagnation Temperatures

The LP CFV stagnation temperature in Stage 2 is measured using a Rosemount 162N100 A resistance temperature device (RTD). This transducer has a probe length of 40 cm thereby allowing sufficient penetration distance into the 76 cm pipe diameter. The uncertainty components for this transducer include the uncertainty of the thermistor transfer standard, (1.2 mK); uniformity of the temperature bath, (1 mK); calibration fit residuals, (10 mK); manufacturer’s drift limit, (20 mK); heat transfer effects (i.e., self heating, stem conduction, and radiation), (10 mK); an estimated spatial sampling error, (60 mK); data acquisition, (30 mK); and the dynamic temperature correction (i.e., temperature adjustment to account for moving gas stagnating against the probe), (0.1 mK). These components are itemized in Table 7. The RSS total for all of the temperature components yields a total temperature uncertainty of 71 mK at *k* = 1.

#### MP CFV Stagnation Temperature

The MP CFV stagnation temperature in Stage 2 is also measured using a Rosemount 162N100A resistance temperature device (RTD). Since the uncertainty components are comparable to those listed in Table 7, they are not repeated here. The total uncertainty equals 71 mK for *k* = 1.

#### Diameter of the LP CFV

The uncertainty in the LP CFV throat diameter is solely attributed to thermal expansion. When the wall throat temperature of LP CFV differs from Stage 1 to Stage 2, the throat diameter will also differ. The change in the throat diameter size with temperature is modeled using the following expression

$$d_{\text{LP}2}=d_{\text{LP}1}\left[1+\alpha \Delta T_{12}\right]$$ (12)

where *d*_LP1_ is the throat diameter during the Stage 1 calibration, *α* = (170 ± 7) × 10^−7^ K^−1^ is the linear coefficient of thermal expansion for stainless steel, and Δ*T*_12_ is change in the throat wall temperature between Stages 1 and 2. Differences in throat wall temperature between Stages 1 and 2 will predominantly be caused by a difference in the respective stagnation temperatures. The wall temperature difference can be estimated by the following formula, $\Delta T_{12}\approx \left(T_{0_{2}}-T_{0_{1}}\right)/\left[1+0.5r\left(\gamma -1\right)\right]$, where *r* = *Pr*^1/3^ is the recovery factor for turbulent flow [5], and $\left(T_{0_{2}}-T_{0_{1}}\right)$ is the difference in the stagnation temperatures between Stages 1 and 2. In this work, a heat exchanger is used to control the Stage 2 stagnation temperature to within 2 K of its Stage 1 value. We estimate the uncertainty of Δ*T*_12_ is 1 K with *k* = 1 so that the uncertainty attributed to thermal expansion of the throat diameter is 17 × 10^−6^.

#### Diameter of the MP CFV

The uncertainty of the throat diameter of the MP CFV is treated analogously to the LP CFV. As a result, the uncertainty is identically zero during its Stage 2 calibration as indicated in Table 4.

#### Critical Flow Function of the LP CFV

The nominal operating conditions of the LP CFV are nearly identical between its calibration in Stage 1 and its use as a transfer standard in Stage 2. As a result, the nominal value of the Stage 1 critical flow function is identical to its Stage 2 counterpart. As explained previously in Stage 1, the net uncertainty contribution is identically zero as observed in Table 4.

#### Critical Flow Function of the MP CFV

The nominal operating conditions of the MP CFV are identical between its calibration in Stage 2 and its use as a transfer standard in Stage 3. As a result, the nominal value of the Stage 2 critical flow function is identical to its Stage 3 counterpart. This methodology is completely analogous to the treatment of the uncertainty for the LP CFV. Therefore, the uncertainty is identically zero in both Stages 2 and 3 as shown in Table 4 (and later in Table 8).

#### Stage 2 Line Packing Effect

The rate of mass storage in the connecting volume between the LP CFVs and the MP CFV is estimated by monitoring the drift in the measured pressure and temperature during the data collection interval. The uncertainty attributed to the line packing effect is estimated by the following expression

$$\frac{\Delta {\dot{m}}_{2}}{{\dot{m}}_{\text{MP}2}}=\left(\frac{M_{\text{st}}/\tau }{{\dot{m}}_{\text{MP}2}}\right)\left|\frac{\delta P_{\text{drift}}}{P}-\frac{\delta T_{\text{drift}}}{T}\right|,$$ (13)

where *M*_st_ is the mass stored in the connecting volume; *τ* is the duration of the test; δ*P*_drift_ and δ*T*_drift_ are the estimated drift in pressure and temperature during the test; and *P* and *T* are the respective pressure and temperature measurements measured at the LP CFV. In the worst case, pressure and temperature drift are in opposite directions. We estimate their magnitudes to be 2 kPa and 0.5 K, respectively. Based on these estimates, the relative uncertainty attributed to the line packing effect is 221 × 10^−6^ for a connecting volume of 2 m^3^ and a 120 s test period. This is a conservative estimate since we expect that both the drift rates and the connecting volume will be smaller when the experiment is conducted and the drift of pressure and temperature may partially cancel.

## 5. Calibration of the HP CFV

### 5.1 Stage 3 Uncertainty Budget

In Stage 3 the MP CFVs are configured in parallel and used to calibrate 21 HP CFVs in dry air. Each one of the HP CFVs is calibrated by itself at nominally the same *P*_0_ and *T*_0_. The calibration setup is similar to the Stage 2 setup shown in Fig. 3, but in this case the HP CFVis positioned upstream of a parallel array of four MP CFVs. Choked flow conditions are verified experimentally using the pressure independence test described in Stage 1. The flow conditions at the MP CFVs are controlled so that their Reynolds numbers (nominally 7.51 × 10^6^ for each venturi) and therefore their discharge coefficients are the same between Stages 2 and 3. Similar to the result in Stage 2, slight mismatches in the Reynolds number have a negligible effect on the venturi discharge coefficient. In Stage 3 the HP CFV stagnation pressure is approximately 16 times the Stage 1 stagnation pressure, and the Reynolds number equals 2.7 × 10^7^.

#### Calibration Procedure

The calibration procedure begins by establishing steady-state flow conditions at the desired *P*_0_ and *T*_0_. Data is then collected for approximately 120 s. The expression for the discharge coefficient of the HP CFV is analogous to the expression for the MP CFV given in Eq. (8). The HP CFV discharge coefficient is

$$C_{d}^{\text{HP}}=\sqrt{\frac{T_{0_{\text{HP}3}}}{T_{0_{\text{MP}3}}}}\left(\frac{P_{0_{\text{MP}3}}C_{\text{MP}3}^{*}}{P_{0_{\text{HP}3}}C_{\text{HP}3}^{*}}\right)\sum_{n=1}^{N_{3}}\left(\frac{d_{{\text{MP}3}_{n}}^{2}}{d_{\text{HP}3}^{2}}\right)C_{d_{n}}^{\text{MP}}$$ (14)

where *N*_3_ = 4 is the number of MP CFVs in parallel. As in Stage 2 the line packing effect is omitted when calculating the discharge coefficient, but is taken into account in the uncertainty analysis. The expression for the HP discharge coefficient in Eq. (14) is the instantaneous value that is recorded during the data collection interval while the reported values are averaged over the collection interval.

#### Expression of Uncertainty for the HP CFV Discharge Coefficient

The relative uncertainty of the HP CFV discharge coefficient is determined by applying the law of propagation of uncertainty to Eq. (14), thereby yielding

$$\begin{array}{l} {\left[\frac{u\left(C_{d}\right)}{C_{d}}\right]}_{\text{HP}}^{2}=\left(\frac{1+r_{\text{MP}}\left(N_{3}-1\right)}{N_{3}}\right){\left[\frac{u\left(C_{d_{n}}\right)}{C_{d_{n}}}\right]}_{\text{MP}}^{2}\\ \;+{\left[1+{\left(\frac{P_{0}}{C^{*}}\frac{\partial C^{*}}{\partial P_{0}}\right)}_{\text{HP}}\right]}^{2}{\left[\frac{u\left(P_{0}\right)}{P_{0}}\right]}_{\text{HP}}^{2}\\ \;+{\left[\frac{1}{2}-\left(\frac{T_{0}}{C^{*}}\frac{\partial C^{*}}{\partial T_{0}}\right)\right]}_{\text{HP}}^{2}{\left[\frac{u\left(T_{0}\right)}{T_{0}}\right]}_{\text{HP}}^{2}\\ \;+{\left[\frac{u\left(P_{0}\right)}{P_{0}}\right]}_{\text{MP}}^{2}+\frac{1}{4}{\left[\frac{u\left(T_{0}\right)}{T_{0}}\right]}_{\text{MP}}^{2}\\ \;+{\left[\frac{u\left(C^{*}\right)}{C^{*}}\right]}_{\text{HP}}^{2}+{\left[\frac{u\left(C^{*}\right)}{C^{*}}\right]}_{\text{MP}}^{2}+4{\left[\frac{u\left(d\right)}{d}\right]}_{\text{HP}}^{2}\\ \;+4{\left[\frac{u\left(d\right)}{d}\right]}_{\text{MP}}^{2}+{\left[\frac{\Delta {\dot{m}}_{3}}{{\dot{m}}_{\text{HP}}}\right]}^{2} \end{array}$$ (15)

Here the correlation coefficient for the parallel array of MP CFVs, *r*_MP_ = 0.88, is computed using the methodology described for Stage 2, and the normalized sensitivity coefficients for [*u*(*P*_0_)]_HP_ and [*u*(*T*_0_)]_HP_ include the pressure and temperature derivatives of C^*^. These additional terms take into account the uncertainty in C^*^ attributed to uncertainties in the stagnation conditions. At the lower pressures in Stages 1 and 2 these additional terms made a negligible contribution to the uncertainty and were omitted. However, at the elevated pressures in Stages 3 through 5 they are not negligible and are therefore included. These derivative terms are approximated using finite differences.

Using Eq. (15) the relative standard uncertainty of the HP CFV discharge coefficient equals 0.086 % (*k* = 1). An itemized list of the uncertainty components is given in Table 8. The largest uncertainty contribution derives from the calibration of the four MP CFVs. The value for this uncertainty (713 × 10^−6^) is slightly less than the value listed previously in Table 4 because the correlation coefficient is less than unity, *r*_MP_ < 1. The uncertainty estimates for remaining uncertainty components are discussed below.

#### Stage 3 MP CFV Stagnation Pressure

The same transducer is used to measure the stagnation pressure of the MP CFV both during its calibration in Stage 2 and its application in Stage 3 (see Table 6). The nominal pressure is also the same in both stages. Consequently, the bias uncertainty components cancel between Stages 2 and 3. The resulting relative uncertainty equals its Stage 2 value of 190 × 10^−6^ for *k* = 1.

#### Stage 3 HP CFV Stagnation Pressure

The stagnation pressure of the HP CFV is measured using a Mensor 11900 DPG pressure transducer. The transducer is calibrated over a 10:1 pressure range that extends from 1720 to 17240 kPa. Similar to the Mensor used to measure *P*_0_ for the MP CFV, the HP CFV Mensor is calibrated every 90 d using a Ruska model 2400H piston assembly with a manufacturer specification of 100 × 10^−6^ at an assumed 95 % confidence level (*k* = 2).

The calibration history for the Mensor spans 9.5 years and includes 348 data points. Based on its calibration history, the short term random uncertainty equals 198 × 10^−6^ while the long term random uncertainty equals 183 × 10^−6^ both at the *k* = 1 level. The uncertainty attributed to the voltage measurement using the Agilent 349070 A data acquisition system is 45 × 10^−6^, and the uncertainty attributed to calculating the stagnation pressure from the static pressure measurement is negligible. The RSS of all of these components yields a total uncertainty of 278 × 10^−6^ at *k* = 1.

#### Stage 3 MP and HP CFV Stagnation Temperatures

The stagnation temperatures at the MP CFV and at the HP CFV in Stage 3 are measured using the same pair of Rosemount 162N100 A temperature transducers that were used in Stage 2. The standard uncertainty for these transducers equals 71 mK at *k* = 1 as given previously in Table 7. The nominal stagnation temperatures at the MP and HP CFV’s are 293 K and 295 K, respectively. Based on these temperatures the standard relative uncertainty for the MP and HP CFV’s are 244 × 10^−6^ and 242 × 10^−6^, respectively.

#### Throat Diameter of the MP CFV

The variation in the throat size of the MP CFV with temperature is given by

$$d_{\text{MP}3}=d_{\text{MP}2}\left[1+\alpha \Delta T_{23}\right]$$ (16)

where *d*_MP2_ is the nominal throat diameter during the Stage 2 calibration, and Δ*T*_23_ is change in the throat wall temperature between Stages 2 and 3. The temperature change is expected to be less than 2 K, but we take Δ*T*_23_ = 2 K ± 1 K. The resulting uncertainty equals 17 × 10^−6^.

#### Throat Diameter of the HP CFV

As discussed in Stage 1, there is no uncertainty attributed to the throat diameter during the calibration stage. Therefore, the throat diameter uncertainty of the HP CFV is identically zero during its Stage 3 calibration.

#### Critical Flow Function of the MP CFV

Because the MP CFV operating conditions and working fluid are nearly identical between Stages 2 and 3, the uncertainty components of the critical flow function are perfectly correlated. As a result, these components contribute a net zero uncertainty as shown in Table 8 and previously in Table 4.

#### Critical Flow Function of the HP CFV

The HP CFV is calibrated in dry air in Stage 3, but used in Stage 4 with natural gas. Because of the change in the working fluid, the assumption of perfect correlation does not apply. Instead we must estimate the three components of uncertainty discussed previously in Stage 1. The procedure used to estimate these components is discussed next.

The critical flow function is a parameter designed to correct for real gas effects. However, it is developed under the assumption of one-dimensional inviscid venturi flow. Consequently, the critical flow function only corrects for real gas behavior in the inviscid core of a one-dimensional nozzle flow. Thus, it does not account for the effect that real gas behavior will have on the sonic line curvature or on the boundary layer. At low pressures, these effects are usually negligible, but become more significant at higher pressures when real gas effects are more significant. A rough estimate of these effects can be made using already developed analytical models [19–21]. Together, these models give first order predictions of the discharge coefficient [22]. The effect of interest here is a second order effect. It is estimated by multiplying the reduction in the discharge coefficient caused by viscous effects with the reduction (or gain) in the discharge coefficient caused by real gas behavior. The result of this multiplication is assumed to have a rectangular distribution and is therefore divided by $\sqrt{3}$ so that the estimated uncertainty equals 135 × 10^−6^ at *k* = 1.

The second component is associated with the thermodynamic database used to calculate C*. Uncertainties in the thermodynamic properties will cause uncertainties in the adiabat necessary for computing C*. We estimated this uncertainty by comparing values of C* as given by Sullivan for dry air [23] with values of C* computed using NIST’s thermodynamic database [24]. The value obtained from this comparison is assumed to have a rectangular distribution so that the resulting uncertainty is 203 × 10^−6^ for *k* = 1.

The third uncertainty component is attributed to the purity of the dry air mixture. Here the major concern is the concentration of water vapor in the mixture. Because the water vapor concentration is sufficiently low (i.e., less than 1 % relative humidity) the uncertainty in attributed to this source is negligible. Thus, the total uncertainty in is obtained by a RSS of the first two uncertainty components, yielding a value of 244 × 10^−6^ for *k* = 1.

#### Line Packing Effect

The line packing effect is estimated using the same approach used in Stage 2. In this case the pressure is measured at the MP CFV. The same values of drift, connecting volume, and collection time are used as in Stage 2. Because the pressure is larger, the mass stored, *M*_st_, is also larger. However this is offset by the increased mass flow ${\dot{m}}_{\text{HP}3}$. On the other hand, the larger pressure at the same drift rate reduces the ratio *δP*_drift_/*P* so that the line packing effect is slightly less than the Stage 2 value. It is calculated to have an uncertainty of 108 × 10^−6^ for *k* = 1.

## 6. Calibration of the TMS Using the Nozzle Bank

### 6.1 Stage 4 Uncertainty Budget

In Stage 4 all nine TMSs are individually calibrated *in their place of use* and *at their normal operating conditions* using natural gas as a working fluid. Each TMS is calibrated using a nozzle bank consisting of the 21 HP CFVs that were calibrated in dry air in Stage 3. The air based calibration can be applied to nozzles flowing natural gas by accounting for real gas effects via the critical flow function, and by matching the Reynolds number. There are no other known specie effects that influence the performance of CFVs at high Reynolds numbers. Because the viscosity of natural gas is less than dry air, matching the Reynolds number requires that *P*_0_ in the natural gas flow be approximately 20 % lower than its value in dry air.

**Fig. 4:**
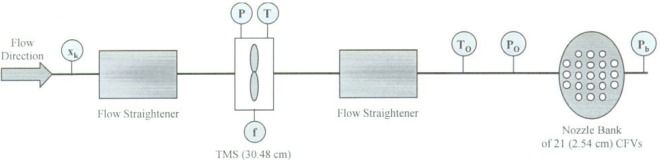
Configuration for calibrating a 30.48 cm TMS using a nozzle bank of twenty-one 2.54 cm CFVs mounted on a 76.2 cm pipeline.

Figure 4 shows a schematic of the calibration setup along with the auxiliary measurements required to determine volumetric flow. Flow in the 76.2 cm diameter pipeline is conditioned using two flow straighteners, one upstream of the TMS and the other upstream of the nozzle bank. As shown in the figure, the static pressure and temperature are measured at the TMS while the stagnation pressure and temperature are measured just upstream of the nozzle bank. Other pertinent measurements include the turbine meter frequency, *f*, and the amount of substance fraction, *x_k_* of the natural gas. All of these measurements are used in conjunction to determine the TMS metering performance.

Each TMS is calibrated over a flow range extending from 0.22 m^3^/s (470 cfm) to 1.22 m^3^/s (2592 cfm). The flow is varied by using control valves to regulate the flow paths through a selected set of the 21 CFVs in nozzle bank. The minimum flow is achieved using just two CFVs and the maximum flow is achieved using only 11 of the 21 CFVs. The additional CFVs in the nozzle bank provide the flexibility to calibrate higher flows if this becomes necessary in the future.

#### Calibration Procedure

The calibration procedure begins by establishing steady-state flow conditions at the desired nominal volumetric flow. Data is then collected for approximately 120 s. A pressure independence test is used to ensure that the nozzle bank remains choked throughout the data collection. At any instant during the data collection the TMS volumetric flow is determined by the following expression

$$q_{\text{TMS}}=\sum_{i=1}^{N_{4}}\frac{{\left({\dot{m}}_{\text{th}}C_{d}\right)}_{i}}{\rho_{\text{TMS}}}+\frac{\Delta {\dot{m}}_{4}}{\rho_{\text{TMS}}}$$ (17)

where *N*_4_ equals the number of CFVs in use, ${\dot{m}}_{{\text{th}}_{i}}=\pi P_{0}d_{i}^{2}C_{\text{gas}}^{*}/\sqrt{16T_{0}R_{u}/{ℳ}_{gas}}$ and $C_{d_{i}}$ are, respectively, the theoretical mass flow and the discharge coefficient of the *i*th CFV in the nozzle bank. In the expression for the theoretical mass flow, $C_{gas}^{*}$ is the natural gas critical flow function. The discharge coefficient for each nozzle is obtained from its Stage 3 calibration at the matched Reynolds number. The density of natural gas at the TMS is given by *ρ*_TMS_ = [*PM*_gas_ *ZR*_u_*T*]_TMS_ where *Z* = *Z*(*P*,*T*,*x_k_*) is the compressibility factor for natural gas, and, ${ℳ}_{\text{gas}}=\sum_{k=1}^{K}x_{k}M_{k}$, is the mixture molecular mass which is equal to a linear sum of the amount of substance fraction, *x_k_*, multiplied by the specie’s molecular weight, *M_k_*. Finally, $\Delta {\dot{m}}_{4}$ 4 is the rate of mass storage in the connecting volume between the TMS and the nozzle bank.

In this work, the calibration performance for each TMS is determined by its *K*-factor, as defined by *K* ≡ *f/q*_TMS_. By substituting the expression for volumetric flow given in Eq. (17) into the definition of the *K* factor we obtain the following formula

$$K=\frac{f}{\frac{\pi }{4}\sqrt{\frac{R_{u}}{{ℳ}_{\text{gas}}}}{\left(\frac{P_{0}C_{\text{gas}}^{*}}{\sqrt{T_{0}}}\right)}_{\text{NB}}{\left(\frac{ZT}{P}\right)}_{\text{TMS}}\left[\sum_{i=1}^{N_{4}}{\left(d_{i}^{2}C_{d_{i}}\right)}_{\text{NB}}\right]}$$ (18)

where the subscripts TMS and NB (i.e., nozzle bank) denote the location of the pressure and temperature measurements. Note that $\Delta {\dot{m}}_{4}$ 4 is set to zero in the calibration equations as in previous stages. The expression for the *K*-factor in Eq. (18) is the instantaneous value that is recorded during the data collection interval. The reported values are averaged over the measurement interval.

#### Expression of Uncertainty for the K-Factor

The uncertainty in the *K*-factor is determined by applying the law of propagation of error to Eq. (18). The resulting expression for relative uncertainty is

$$\begin{array}{l} {\left[\frac{u\left(K\right)}{K}\right]}^{2}=\left(\frac{1+r_{\text{NB}}\left(N_{4}-1\right)}{N_{4}}\right){\left[\frac{u\left(C_{d}\right)}{C_{d}}\right]}_{\text{NB}}^{2}+{\left[\frac{u\left(C^{*}\right)}{C^{*}}\right]}_{\text{gas}}^{2}\\ \;+{\left[\frac{u\left(Z\right)}{Z}\right]}_{\text{TMS}}^{2}+\left(\frac{3}{4}\right){\left[\frac{u\left(R_{u}\right)}{R_{u}}\right]}^{2}+4{\left[\frac{u\left(d_{p}\right)}{d_{p}}\right]}_{\text{NB}}^{2}\\ \;+{\left[\frac{u\left(f\right)}{f}\right]}^{2}+u_{\text{tot}}^{2}\left(x_{k}\right)+u_{\text{tot}}^{2}\left(M_{k}\right)+u_{\text{tot}}^{2}\left(P\right)+u_{\text{tot}}^{2}\left(T\right)\\ \;+{\left[\frac{\Delta {\dot{m}}_{4}}{\rho_{\text{TMS}}\;q_{\text{TMS}}}\right]}^{2} \end{array}$$ (19)

where the terms $u_{\text{tot}}^{2}\left(x_{k}\right),u_{\text{tot}}^{2}\left(P\right),u_{\text{tot}}^{2}\left(T\right)$, and $u_{\text{tot}}^{2}\left(M_{k}\right)$ are interim variables, that group together like uncertainty terms. For example, *u*_tot_(*P*) is the combined pressure uncertainty for both the TMS and the nozzle bank. The definitions of these four terms are given below. The correlation coefficient, *r*_NB_ = 0.87, was computed using the methodology described in Stage 2. The *K*-factor uncertainty decreases slightly with increasing volumetric flow. At the lowest flow (i.e., *N*_4_ = 2), the uncertainty equals 1444 × 10^−6^ at *k* = 1. At the highest flow (i.e., *N*_4_ = 11) the uncertainty decreases to 1420 · 10^−6^ at *k* = 1. An itemized list of all of the uncertainty components is provided in Table 9 for the lowest flow where *q*_TMS_ = 0.22 m^3^/s and *N*_4_ = 2.

#### Discharge Coefficient of the CFVs in the Nozzle Bank

As expected, the largest source of uncertainty derives from the *C*_d_ values of the CFVs in the nozzle bank. The magnitude of this uncertainty is determined from the Stage 3 calibration of the HP CFVs. The value for the array of *N*_4_ = 2 CFVs in parallel given in Table 9 (830 × 10^−6^) is slightly less than the value for a single CFV given previously in Table 8. This is a consequence of the CFVs in the nozzle bank having a correlation coefficient that is less than unity, *r*_NB_ < 1.

#### Critical Flow Function for the Nozzle Bank

During the calibration of the TMS the nominal stagnation pressure at the nozzle bank is 7175 kPa. In natural gas flows at this high pressure, real gas effects substantially affect the performance of CFVs. Correlations for real gas effects are made via $C_{\text{gas}}^{*}$, the real gas critical flow function. The uncertainty components for $C_{\text{gas}}^{*}$ are shown in Table 10. The real gas effects on the sonic line and boundary layer (419 × 10^−6^) are estimated using the methodology discussed in the Stage 3 analysis. The uncertainty attributed to the thermodynamic database used to calculate $C_{\text{gas}}^{*}$ (577 × 10^−6^) was estimated by comparing the results from [23] with values computed using the NIST thermodynamic database [24]. The resulting uncertainty for the critical flow function is 713 × 10^−6^ at = 1.

Typically, the values of $C_{\text{gas}}^{*}$ are calculated as a function of *P*_0_ and *T*_0_ for a given specie. Therefore, uncertainties in the measurement of these variables will affect the uncertainty in $C_{\text{gas}}^{*}$, and ultimately the uncertainty in the *K*-factor. These types of uncertainties are taken into account in the sensitivity coefficients that multiply the respective uncertainties, *u*(*P*_0_), *u*(*T*_0_), and *u*(*x_k_*).

#### TMS Compressibility Factor

The compressibility factor for natural gas is determined using the AGA 8 Thermodynamic Database [25]. The database specifies a relative uncertainty of 1000 × 10^−6^. Assuming a rectangular distribution, we divide this value by $\sqrt{3}$ to obtain an relative uncertainty of 577 × 10^−6^.

#### Composition Analysis

Measurements of the gas composition are necessary to determine the TMS *K*-factor. Although gas composition does not explicitly appear in the determination of the *K*-factor [see (18)], the gas composition is necessary to determine the compressibility factor, *Z*_TMS_; the molecular mass, ℳ_gas_; and the critical flow function, $C_{\text{gas}}^{*}$. As such, these variables are reflected as sensitivity coefficients in the expression of the total uncertainty in gas composition as given by

$$u_{\text{tot}}^{2}\left(x_{k}\right)=\sum_{k=1}^{K}{\left[\frac{1}{2}\left(\frac{x_{k}M_{k}}{{ℳ}_{\text{gas}}}\right)-\left(\frac{x_{k}}{C_{\text{gas}}^{*}}\frac{\partial C_{\text{gas}}^{*}}{\partial x_{k}}\right)-\left(\frac{x_{k}}{Z_{\text{TMS}}}\frac{\partial Z_{\text{TMS}}}{\partial x_{k}}\right)\right]}^{2}{\left[\frac{u\left(x_{k}\right)}{x_{k}}\right]}^{2}$$ (20)

Among the three terms in the sensitivity coefficients, the molecular weight is the most important. It is almost an order of magnitude larger than the other two. For completeness, however, the critical flow function and the compressibility factor are included in the analysis. These derivative terms are evaluated numerically by using finite differences.

In Stage 4, gas chromatography is used to measure the mole fraction of each component. A sample of natural gas will be collected during the calibration of each TMS for later analysis by the Gas Metrology and Classical Methods Group at NIST. Table 11 gives the nominal value of the mole fractions of natural gas at the CEESI Iowa facility as well as their expected uncertainties at the 95 % confidence level. Based on these values, the total uncertainty in gas composition equals *u*_tot_(*x_k_*) = 467 × 10^−6^.

#### Frequency of the TMS

The frequency of the TMS is measured using an Agilent 53131A counter over a range extending from 50 Hz to 250 Hz. Assuming a trigger level error of 0.5 % of the signal period and a gate time of 100 s, the manufacturer specifications for the uncertainty is below 10 × 10^−6^ over the entire frequency range. This value is assumed to be stated at a 95 % confidence level (*k* = 2). Thus, the relative uncertainty in frequency is taken equal to be [*u*(*f*)/*f*] = 5 × 10^−6^ for *k* = 1.

#### Measurements of the Nozzle Bank Stagnation Pressure and TMS Pressure

In Stage 4 the pressure measurements are made with four Rosemount 3051 pressure transducers. Two of these transducers are used to measure the pressure at the TMS while the remaining two measure the nozzle bank stagnation pressure. The redundancy of using two transducers safeguards against erroneous pressure readings. Table 12 shows the uncertainty components of a typical Rosemount pressure transducer. These transducers are calibrated every 90 d using an Ametek EPC 2000 pressure transducer. The relative uncertainty of the Ametek transfer standard is 290 × 10^−6^ over a pressure range extending from 6200 kPa to 8300 kPa. The Ametek is calibrated at six month intervals using a Ruska dead weight tester. Based on the calibration history of the Rosemount transducers, typical values for the short term and long term random effects are computed to be 160 × 10^−6^ and 100 × 10^−6^, respectively. Finally, the data acquisition uncertainty attributed to the voltage measurement is 31 × 10^−6^.

The uncertainty of the TMS pressure measurements are determined by a RSS of the four uncertainty components in Table 12 so that [*u*(*P*)/*P*]_TMS_ = 347 × 10^−6^. The uncertainty of the nozzle bank (NB) stagnation pressure also includes the four components given in Table 12 plus an additional component (26 × 10^−6^) attributed to calculating *P*_0_ from the static pressure measurement [see Eq. (6)]. Thus, the uncertainty in the NB stagnation pressure is slightly larger, equaling [*u*(*P*_0_)/*P*_0_]_NB_ = 348 × 10^−6^ for *k* = 1.

The total uncertainty in pressure, *u*_tot_(*P*), includes measurements at both the TMS and at the nozzle bank. However, *u*_tot_(*P*) is not equal to the RSS of these components. Because the pressure transducers are traceable to the same calibration standard, the bias errors associated with the calibration are perfectly correlated. When this correlation is considered, the total pressure uncertainty is given by

$$\begin{array}{l} u_{\text{tot}}^{2}\left(P\right)={\left[1+\left(\frac{P_{0}}{C_{\text{gas}}^{*}}\frac{\partial C_{\text{gas}}^{*}}{\partial P_{0}}\right)\right]}^{2}{\left[\frac{u\left(P_{0}\right)}{P_{0}}\right]}_{\text{NB}}^{2}\\ \;+{\left[1-{\left(\frac{P}{Z}\frac{\partial Z}{\partial P}\right)}_{\text{TMS}}\right]}^{2}{\left[\frac{u\left(P\right)}{P}\right]}_{\text{TMS}}^{2}\\ \;-2\left[1+\left(\frac{P_{0}}{C_{\text{gas}}^{*}}\frac{\partial C_{\text{gas}}^{*}}{\partial P_{0}}\right)\right]\left[1-{\left(\frac{P}{Z}\frac{\partial Z}{\partial P}\right)}_{\text{TMS}}\right]\\ \;{\left[\frac{u_{c}\left(P_{0}\right)}{P_{0}}\right]}_{\text{NB}}{\left[\frac{u_{c}\left(P\right)}{P}\right]}_{\text{TMS}} \end{array}$$ (21)

where [*u*c(*P*_0_)/*P*_0_]_NB_ and [*u*c(*P*)/*P*]_TMS_ are the correlated uncertainty components associated with their calibration by the Ametek EPC 2000. The relative standard uncertainty of both of these correlated uncertainty components is 290 × 10^−6^ as shown in Table 12. Using Eq. (21) the total relative pressure uncertainty equals *u*_tot_(P) = 297 × 10^−6^ for *k* = 1.

#### Measurements of the Nozzle Bank Stagnation Temperature and TMS Temperature

In Stage 4 the temperature measurements at the nozzle bank and at the TMS being calibrated are made using a set of Rosemount 3144 RTD’s. Temperature redundancy is accomplished using two RTD’s at both the TMS and at the nozzle bank. Consequently, each temperature measurement is the average of two RTD readings.

The nominal value for the uncertainty components of a Rosemount 3144 RTD is specified in Table 13. A Hart Scientific 1521 is used as the temperature transfer standard. It has a manufacturer specified relative uncertainty of 98 × 10^−6^ (*k* = 1). The remaining uncertainty components of the RTD include the following: digital accuracy (200 × 10^−6^), manufacturer-specified drift limit (198 × 10^−6^), ambient temperature effects (127 × 10^−6^), data acquisition system (6 × 10^−6^), and probe heat transfer effects (50 × 10^−6^). All but the probe heat transfer effects are determined based on manufacturer’s specifications. The total uncertainty of the TMS temperature using this RTD equals [*u*(*T*)/*T*]_TMS_ = 328 × 10^−6^. The uncertainty of the nozzle bank stagnation temperature has an additional uncertainty component attributed to the correction in calculating *T*_0_ from the measured static temperature. However, this correction is small enough to be considered negligible so that [*u*(*T*)/*T*]_NB_= 328 × 10^−6^ as well.

We expect that the temperatures at the nozzle bank and at the TMS to be nominally the same. Since the RTD’s are all traceable to the same temperature standard, calibration biases are identical among them. Here, we take these bias components to be perfectly correlated so that the total temperature uncertainty is given by the following relationship

$$\begin{array}{l} u_{\text{tot}}^{2}\left(T\right)={\left[\frac{1}{2}-\left(\frac{T_{0}}{C_{\text{gas}}^{*}}\frac{\partial C_{\text{gas}}^{*}}{\partial T_{0}}\right)\right]}^{2}{\left[\frac{u\left(T_{0}\right)}{T_{0}}\right]}_{\text{NB}}^{2}\\ \;+{\left[1+\left(\frac{T}{Z}\frac{\partial Z}{\partial T}\right)\right]}^{2}{\left[\frac{u\left(T\right)}{T}\right]}_{\text{TMS}}^{2}\\ \;-2\left[\frac{1}{2}-\left(\frac{T_{0}}{C_{\text{gas}}^{*}}\frac{\partial C_{\text{gas}}^{*}}{\partial T_{0}}\right)\right]\left[1+\left(\frac{T}{Z}\frac{\partial Z}{\partial T}\right)\right]\\ \;{\left[\frac{u_{c}\left(T_{0}\right)}{T_{0}}\right]}_{\text{NB}}{\left[\frac{u_{c}\left(T\right)}{T}\right]}_{\text{TMS}} \end{array}$$ (22)

In this expression the correlated components of uncertainty, [*u*_c_(*T*_0_)]_NB_ and [*u*_c_(*T*)]_TMS_, both equal the calibration bias (28 mK). Using these values in Eq. (22) yields a total relative temperature uncertainty *u*_tot_(*T*) = 447 × 10^−6^ at *k* = 1.

#### Throat Diameter of CFVs in the Nozzle Bank

The nozzle bank consists of the 21 HP CFVs that were calibrated in Stage 3. The variation in the throat size for anyone of the HP CFVs in the nozzle bank is

$$d_{\text{NB}}=d_{\text{HP}3}\left[1+\alpha \Delta T_{34}\right]$$ (23)

where Δ*T*_34_ is change in the throat wall temperature between Stages 3 and 4. This temperature change is expected to be well less than 10 K. As an estimate we take Δ*T*_34_ = (10 ± 2) K so that the uncertainty equals 69 × 10^−6^.

#### Molecular Mass of Individual Constituents

The mixture molecular weight, ${ℳ}_{\text{gas}}=\sum_{k=1}^{K}x_{k}M_{k}$, depends on both the specie composition, *x_k_*, and on the molecular weight of each component in the mixture, *M_k_*. The uncertainty attributed to specie composition has already been considered. Here, we consider the uncertainty attributed to the molecular weight of each component. The total uncertainty due to the contribution of all the individual constituents is given by the following formula

$$u_{\text{tot}}^{2}\left(M_{k}\right)=\frac{1}{4}\sum_{k=1}^{K}{\left(\frac{x_{k}M_{k}}{{ℳ}_{\text{gas}}}\right)}^{2}{\left[\frac{u\left(M_{k}\right)}{M_{k}}\right]}^{2}.$$ (24)

Given that the molecular weight of individual constituents is known to better than 1 × 10^−6^, the total uncertainty contribution from the individual molecular weight constituents is less than < 1 × 10^−6^.

#### Universal Gas Constant

The universal gas constant has a value of *R_u_* = 8314.472 J/(kg ⋅ K) with a relative uncertainty of 2 × 10^−6^ [17].^5^

#### Stage 4 Line Packing Effect

As in Stages 2 and 3, no attempt is made to directly measure the line packing effect. It is estimated by monitoring the drift in the measured pressure and temperature during the data collection interval. We conservatively estimate that the pressure and temperature drift are in opposite directions having magnitudes of 0.2 kPa and 0.2 K, respectively, during the 120 s data collection. Assuming that the connection volume between the TMS and the nozzle bank equals 7.1 m^3^ and the volumetric flow is *q*_TMS_ = 0.22 m^3^/s, the line packing uncertainty is 171 × 10^−6^.

## 7. Calibration of the MUT Using the TMS Array

### 7.1 Stage 5 Uncertainty Budget

**Fig. 5:**
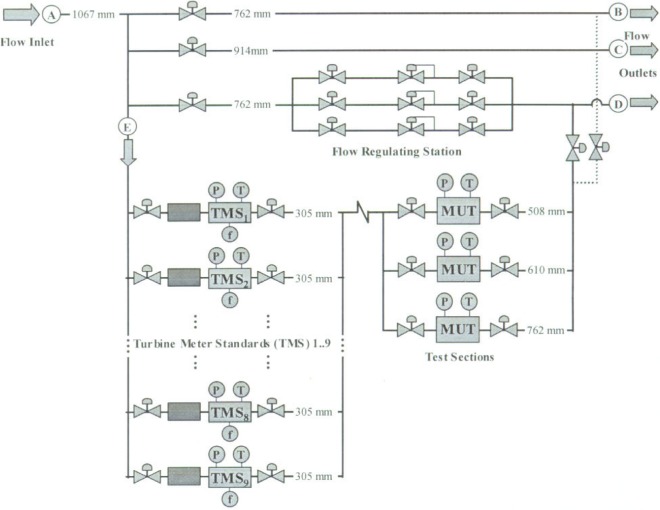
Schematic diagram of the CEESI Iowa Natural Gas Calibration Facility.

The CEESI Iowa calibration facility is shown in Fig. 5. Gas enters the facility through a 1067 mm diameter pipe at A and exits the facility through three pipes labeled B, C, and D having diameters of 762 mm, 914 mm, and 762 mm, respectively. Pipeline E functions as the calibration loop. The nominal volumetric flow through the calibration loop is set using the flow regulation station located on pipeline D. Secondary flow control, if necessary, is accomplished by adjusting control valves in the test section of the calibration loop.

Flow determination through the calibration loop E is based on the parallel array of nine 305 mm (12 in) TMS that were individually calibrated *in their place of use* and *at their normal operating pressure* in Stage 4. One or more of these TMS is used to measure a particular flow. For a given flow measurement, the optimum number of TMS is used to attain the lowest uncertainty. Double block and bleed valves are used to prevent any leakage to or from the TMS not in use. The maximum flow in the test section of 10.7 m^3^/s (22 500 cfm) is attained when all nine turbine meters are operating at full capacity.

Calibrations performed at this facility are conducted at ambient temperatures and at pressures between 6900 kPa and 7600 kPa. The MUT is located downstream of the TMS in one of three test sections having diameters of 508 mm, 610 mm, and 762 mm. Check standards (not shown in Fig. 4) have been installed in each of the three test sections in series with the MUT to help ensure consistent performance of the calibration facility.

#### Theoretical Background for Volumetric flow Determination

The measurement principle for the calibration facility is conservation of mass. The net influx of mass into a specified volume is equal to the rate of mass accumulation or storage in that region. By applying conservation of mass to the region of the calibration loop (i.e., pipeline E) between the TMS and the MUT, the total volumetric flow through the MUT is

$$q_{\text{MUT}}=\sum_{i=1}^{N_{5}}\left(\frac{\rho_{{\text{TMS}}_{i}}}{\rho_{\text{MUT}}}\right)q_{{\text{TMS}}_{i}}-\frac{\Delta {\dot{m}}_{5}}{\rho_{\text{MUT}}}$$ (25)

where *N*_5_ is the number of TMS being used; $\rho_{\text{TMS}}={\left[Pℳ/R_{u}ZT\right]}_{{\text{TMS}}_{i}}$ and $q_{{\text{TMS}}_{i}}={\left[f/K\right]}_{{\text{TMS}}_{i}}$ are the density and actual volumetric flow for the *i*thTMS respectively, *p*_MUT_ = [*P*ℳ/*R*_u_*ZT*]_MUT_ is the density at the MUT, and $\Delta {\dot{m}}_{5}$ is the total rate of mass storage.

Given that the nominal pressure and temperature are the same at both the MUT and at each TMS, the density ratio in Eq. (25) has a value close to unity. Moreover, the uncertainty in the density of the MUT (or any one of the TMS) is substantially larger than the uncertainty of the density ratio. This is a consequence of the correlation between *P*_MUT_ and $P_{{\text{TMS}}_{i}}$. To account for the correlated uncertainties in the density ratio in a straightforward manner, the expressions for *P*_MUT_ and $P_{{\text{TMS}}_{i}}$ are substituted into Eq. (25), to yield

$$q_{\text{MUT}}=\left(\frac{{ℳ}_{\text{TMS}}}{{ℳ}_{\text{MUT}}}\right)\sum_{i=1}^{N_{5}}\left(\frac{\rho_{{\text{TMS}}_{i}}}{\rho_{\text{MUT}}}\right)\left(\frac{T_{\text{MUT}}}{T_{{\text{TMS}}_{i}}}\right)\left(\frac{Z_{\text{MUT}}}{Z_{{\text{TMS}}_{i}}}\right)\left(\frac{f_{{\text{TMS}}_{i}}}{K_{{\text{TMS}}_{i}}}\right),$$ (26)

where the TMS volumetric flow has been expressed as the ratio of frequency to *K*-factor, and the mass storage term is omitted. When using Eq. (26) to compute *q*_MUT_, the ratio of the molecular weights should be set equal to unity. This ratio is explicitly retained in the equation because it has an uncertainty from the drift in the gas composition during a calibration.

#### Expression of Uncertainty for MUT Volumetric Flow

By applying the method of propagation of uncertainty to Eq. (26) the relative uncertainty of the MUT volumetric flow is given by the following expression

$$\begin{array}{l} {\left[\frac{u\left(q\right)}{q}\right]}_{\text{MUT}}^{2}=\left(\frac{1+r_{\text{TMS}}\left(N_{5}-1\right)}{N_{5}}\right){\left[\frac{u\left(K\right)}{K}\right]}_{\text{TMS}}^{2}+{\left[\frac{u\left(f\right)}{f}\right]}_{\text{TMS}}^{2}\\ \;+u_{\text{tot}}^{2}\left(P\right)+u_{\text{tot}}^{2}\left(T\right)+u_{\text{tot}}^{2}\left(x_{k}\right)+u_{\text{tot}}^{2}\left(M_{k}\right)+u_{\text{tot}}^{2}\left(Z\right)\\ \;+{\left[\frac{\Delta m_{5}}{\rho_{\text{MUT}}q_{\text{MUT}}}\right]}^{2} \end{array}$$ (27)

where the correlation coefficient, *r*_TMS_ = 0.86, is calculated using the method discussed in the Stage 2 analysis. For brevity, the total relative uncertainty in pressure, *u*_tot_(*P*); temperature, *u*_tot_(*T*); composition, *u*_tot_(*x_k_*); molecular weight of individual species, *u*_tot_(*Mk*); and the compressibility factor, *u*_tot_(*Z*); have been grouped together into like terms. By definition the normalized sensitivity coefficients of these grouped terms is unity. The relative uncertainty in the MUT volumetric flow ranges from 1428 × 10^−6^ at the highest flow (10.7 m^3^/s) to 1526 × 10^−6^ at the lowest flow (0.65 m^3^/s). The increased uncertainty at low flows is caused by the increased effect of line packing coupled with the increased value of the coefficient that multiplies [*u*(*K*)/*K*]_TMS_ at smaller values of *N*_5_.

#### MUT and TMS Pressures

During a calibration process, pressure measurements both at the MUT and at each TMS in use are made using a pair of Rosemount 3051 transducers. The nominal uncertainty components for these transducers were shown previously in Table 12. The total pressure uncertainty includes the uncertainty of the TMS and the MUT. Because all of the characterizations of these transducers are traceable to the same calibration standard, the bias uncertainty components are assumed to completely cancel and the total relative uncertainty in pressure is given by

$$u_{\text{tot}}^{2}\left(P\right)={\left[1-\left(\frac{P}{Z}\frac{\partial Z}{\partial P}\right)\right]}^{2}\left({\left[\frac{u_{u}\left(P\right)}{P}\right]}_{\text{MUT}}^{2}+\frac{1}{N_{5}}{\left[\frac{u_{u}\left(P\right)}{P}\right]}_{\text{TMS}}^{2}\right)$$ (28)

where the uncorrelated pressure components, [*u*_u_(*P*)/*P*]_MUT_ and [*u*_u_(*P*)/*P*]_TMS_, both equal 191 × 10^−6^. For simplicity, each transducer is assumed to have the same random uncertainties. The random uncertainty components are from the short term random effects (160 × 10^−6^), the long term random effects (100 × 10^−6^), and the data acquisition (31 × 10^−6^). When Eq. (28) is used with these values, the total pressure uncertainty equals *u*_tot_(*P*) = 266 × 10^−6^.

#### TMS and MUT Temperature

The temperatures at the MUT and at each of the TMS are measured using a pair of the Rosemount 3144 RTD’s. These same RTD’s were used in Stage 4 and the uncertainty components for these transducers were given previously in Table 13. The nominal temperature is the same at both the MUT and at the array of TMS. Because the characterizations of these transducers are all traceable to the same temperature transfer standard, bias uncertainty components completely cancel and the total relative uncertainty in temperature is given by

$$u_{\text{tot}}^{2}\left(T\right)={\left[1+\left(\frac{T}{Z}\frac{\partial Z}{\partial T}\right)\right]}^{2}\left({\left[\frac{u_{u}\left(T\right)}{T}\right]}_{\text{MUT}}^{2}+\frac{1}{N_{5}}{\left[\frac{u_{u}\left(T\right)}{T}\right]}_{\text{TMS}}^{2}\right)$$ (29)

where the uncorrelated temperature uncertainty components, [*u*_u_(*T*)/*T*]_MUT_ and [*u*_u_(*T*)/*T*]_TMS_, both equal 313 × 10^−6^. In this case, the uncorrelated uncertainty components are assumed to include the digital accuracy (200 × 10^−6^), the stability (198 × 10^−6^), the ambient temperature effect (127 × 10^−6^), the data acquisition (6 × 10^−6^), and probe heat transfer effects (50 × 10^−6^). Using these values in conjunction with Eq. (29), the total temperature uncertainty equals *u*_tot_(*T*) = 376 × 10^−6^.

#### Species Composition

In Stage 5, the gas composition is measured using an industrial grade gas chromatograph. Unlike the pressure and temperature, which are measured at multiple locations (i.e., at each TMS and at the MUT), the composition is measured at a single location. During a calibration, a sample of gas is collected from pipeline E (see Fig. 5) for later analysis. Only one measurement of the gas composition is necessary since it remains almost constant during a calibration. Furthermore, the uncertainty from this single measurement completely cancels when used to determine the ratios of molecular weight and compressibility factor in Eq. (26). The only uncertainty is due to drift in gas composition during a calibration. Historical gas species data show that the composition of the incoming supply gas drifts over the course of a day. The drift was estimated by statistically analyzing more than a year’s worth of composition data. An expression that gives the relative uncertainty in gas composition attributed to drift is

$$u_{\text{tot}}^{2}\left(x_{k}\right)={\sum_{k=1}^{K}\left(\frac{x_{k}}{Z}\frac{\partial Z}{\partial x_{k}}-\frac{x_{k}}{ℳ}\frac{\partial ℳ}{\partial x_{k}}\right)}^{2}{\left[\frac{\sigma x_{k}}{x_{k}}\right]}^{2}$$ (30)

where $\sigma_{x_{k}}/x_{k}$ is the standard deviation of the drift for each species in the natural gas mixture. Using this expression the total uncertainty in gas composition equals *u*_tot_(*x_k_*) = 208 × 10^−6^.

#### TMS and MUT Compressibility Factor

The compressibility factor is determined at the MUT as well as at each TMS being used. In all cases, the AGA 8 Thermodynamic Database [25] is used to evaluate the compressibility factor. Since the nominal pressure, temperature, and gas composition are the same at the MUT and at the TMS array, the uncertainty resulting from the thermodynamic database completely cancels, yielding a zero uncertainty.

#### Molecular Mass of Individual Constituents

The molecular mass of individual components has a sensitivity coefficient equal to zero. Consequently, it contributes zero uncertainty to the analysis.

##### Frequency of the TMS

The frequency outputs from the TMS are measured using the Agilent 53131A counters discussed in Stage 4. Thus, the uncertainty is the same as in Stage 4, having a value of 10 × 10^−6^ at an assumed 95 % confidence level.

#### Stage 5 Line Packing Effect

As in the previous stages, no attempt is made to directly measure the line packing effect. It is estimated by monitoring the drift in the measured pressure and temperature during the data collection interval. Historical calibration data show that the average drift in pressure and temperature equal 0.5 kPa and 0.4 K during the 120 s data collection, respectively. Assuming that the connecting volume between the TMS and the nozzle bank equals 62.3 m^3^ and the volumetric flow is *q*_MUT_ = 1.3 m^3^/s the relative uncertainty for the line packing effect is 167 × 10^−6^.

## 8. Summary and Conclusions

This paper provides the pathway whereby the results of a MUT calibrated at the CEESI Iowa calibration facility produces results that are traceable to the NIST 26 m^3^ *PVTt* primary calibration standard. This traceability is accomplished in five stages. In Stage 1 four CFVs are calibrated in dry air at low pressure (i.e., 570 kPa) using NIST’s 26 m^3^ *PVTt* primary flow standard. Stages 2 and 3 consist of a pressure ramp-up process (also in dry air) whereby the calibration of the Stage 1 CFVs is transferred to 21 CFVs at high pressure (9200 kPa). In Stage 4, the 21 high pressure CFVs are installed into a nozzle bank and used to calibrate nine TMS at high pressure using natural gas as the working fluid. Finally, in Stage 5, the nine TMS are used to calibrate a MUT over a flow range extending from 0.71 m^3^/s (1500 cfm) to 10.7 m^3^/s (22 460 cfm). The relative expanded uncertainty varies from 0.28 % at the highest flow to 0.3 % at the lowest flow with a coverage factor of *k* = 2.

The method for providing NIST traceability for the measurement results from the CEESI Iowa calibration facility involves using two types of transfer standards, including CFVs and TMS. The CFV flow standard is based on mass flow while the TMS flow standard is based on volumetric flow. The purpose of the CFV standard is to provide traceability for the results when different species are used (e.g., air to natural gas). The effect of gas species at high Reynolds numbers is quantitatively understood for CFVs, but not for turbine meters. Consequently, the CFVs transfer standard allows the air-based NIST calibration to be applied in natural gas flows. Other advantages offered by the nozzle bank of CFVs are the ability to calibrate each TMS *in its place of use* and *at its operating line pressures*. Thus, we avoid not only the potential problems caused by gas species effects, but also problems attributed to installation, and possible pressure effects.

The drawback of using the mass based CFV standard to calibrate a volume based TMS standard is the need to accurately determine both the density (i.e., compressibility factor, gas composition, temperature, etc.) and the critical flow function. In high pressure natural gas flows the uncertainties in determining these quantities are quite large relative to flow measurement. As shown in Table 9 of Stage 4, the uncertainty contribution from *ρ*_gas_ plus $C_{\text{gas}}^{*}$, exceeds 60 %, and thereby introduces more uncertainty in the calibration than the CFV flow standard. This uncertainty could be avoided if species effects in turbine meters were better understood. Future research efforts should focus on quantifying how gas species affect turbine meter performance. Understanding these effects could eliminate the need to determine *ρ*_gas_ and $C_{\text{gas}}^{*}$, thereby significantly reducing the uncertainty.

## Figures and Tables

**Table 1:** Stage 1 uncertainty budget for the discharge coefficient of the LP CFV using dry air

Uncertainty of stage 1 discharge coefficient	Relative uncertainty (*k* = 1)	Normalized sensitivity Coefficient	Percent contribution	Comments
LP CFV Discharge Coefficient, *C*_d_ = 0.9924	(× 10^−6^)	(-----)	(%)	
*PVTt* primary standard, $\left({\dot{m}}_{PVTt}=675.2\;g/s\right)$	650	1.0	95.1	Unc. of *PVTt* at *k* = 1 [2–4]
Stagnation pres. LP CFV, (*P*_0_ = 570.03 kPa)	118	1.0	3.2	See Table 2
Stagnation temp. LP CFV, (*T*_0_ = 296.01 K)	177	0.5	1.7	See Table 3
Throat diameter, (d = 2.54 cm)	0	2.0	0	Nom. value is fixed betw. Stages 1 and 2
Molecular mass, (ℳ = 28.9639 g/mol)	25	0.5	0	See explanation below
Univ. gas constant, (*R*_u_ = 8314.472 J/kmol K)	2	0.5	0	See ref. [17]
Critical flow function, (C^*^ = 0.6864)	0^*^	1.0	0	Same flow cond. in Stages 1 and 2
RSS	667		100	

**Table 2:** Uncertainty of the Stage 1 stagnation pressure

Poroscientific Model 740	Relative uncertainty (*k* = 1)	Absolute uncertainty	Percent contribution	Comments
LP CFV stagnation pres., *P*_0_ = 570.03 kPa	(× 10^−6^)	(Pa)	(%)	
Calibration transfer standard for static pres.	17	9.7	2.1	Traceable to NIST Pres. and Vacuum Group
Drift limit	60	34.2	25.6	< 0.01 % in 6 months, assume rect. distrib.
Residuals, hysteresis, thermal effects	100	57.0	71.3	From cal. records, experiments
Dynamic pres. Unc.	12	6.7	1	Est. based on unc. of 10 % u(M) and 5 %
RSS	118	67.5	100	

**Table 3:** Uncertainty of the Stage 1 stagnation temperature

YSI Model 46000 Thermistor	Relative uncertainty (*k* = 1)	Absolute uncertainty	Percent contribution	Comments
LP CFV stagnation temp., *T*_0_ = 296 K	(× 10^−6^)	(mK)	(%)	
Calibration transfer standard for static temp.	4	1.2	0.1	Traceable to NIST Thermometry Group
Uniformity of temperature bath	3	1.0	0.0	Expt. measurement
Fit residuals	34	10.0	3.7	Based on calibration data
Drift (I, R, DMM, thermistors)	34	10.0	3.7	Manuf. spec. < 10 mK/10 months, rect.
Radiation, stem, cond., self-heating	17	5.0	0.9	Expt. varied current
Spatial sampling error	169	50.0	91.6	Expt. varied insertion depth
Stagnation. vs static	3	1.0	0.0	Est. correction for dynamic flow
RSS	177	52	100	

**Table 4:** Uncertainty in the Stage 2 calibration of the MP CFVs

Uncertainty of stage 2 MP discharge coefficient	Relative uncertainty (*k* = 1)	Normalized sensitivity Coefficient	Percent contribution	Comments
MP CFV discharge coefficient, *C*_d_ = 0.9929	(× 10^−6^)	(-----)	(%)	
Discharge coeff. LP CFV, (*C*_d_*_n_* = 0.9924)	655	1.0	76.5	Stage 1 calibration (corr. effects included)
Stagnation pres. MP CFV, (*P*_0_ = 2280.13 kPa)	190	1.0	6.5	See Table 6
Stagnation pres. LP CFV, (*P*_0_ = 570.03 kPa)	127	1.0	2.9	See Table 5
Stagnation temp. MP CFV, (*T*_0_ = 295.00 K)	242	0.5	2.6	See explanation below
Stagnation temp. LP CFV, (*T*_0_ = 294.00 K)	243	0.5	2.6	See Table 7
Critical flow function MP CFV, (C* = 0.6929)	0*	1.0	0	Perfectly correlated betw. stages 2 and 3
Critical flow function LP CFV, (C* = 0.6869)	0*	1.0	0	Perfectly correlated betw. stages 1 and 2
Diameter MP CFV, (*d* = 2.54 cm)	0	2.0	0	Zero uncertainty during calibration stage
Diameter LP CFV, (*d* = 2.53996 cm)	17	2.0	0.2	Est. unc. for Δ*T*_12_ = 2 ± 1 K
Line packing effect	221	1.0	8.7	Based on expected *T* and *P* drift during cal.
RSS	749		100	

**Table 5:** Stagnation pressure uncertainty of the Stage 2 LP CFVs

Mensor 15000 DPG	Relative uncertainty (*k* = 1)	Absolute uncertainty	Percent contribution	Comments
LP CFV stagnation pres., *P*_0_ = 570.03 kPa	(× 10^−6^)	(Pa)	(%)	
Calibration transfer standard.	75	42.8	35.1	Model RK-300 dead weight tester
Short term random uncertainty	56	31.8	19.5	Statistical process control [18]
Long term andom uncertainty	52	29.9	17.2	Statistical process control [18]
Data Acquisition (Agilent 349070 A)	67	38.3	28.2	Manuf. Spec.
Dynamic pres. Unc.	1	0.5	0	Est. based on Unc. of 10 % *u*(*M*) and 5 % *u*(*γ*)
RSS	127	72.1	100	

**Table 6:** Stagnation pressure uncertainty of the Stage 2 MP CFV

Mensor 11900 DPG	Relatively uncertainty (*k* = 1)	Absolute uncertainty	Percent contribution	Comments
MP CFV stagnation pres., *P*_0_ = 2280 kPa	(× 10^−6^)	(Pa)	(%)	
Calibration transfer standard.	50	114.01	3.7	Ruska piston assembly, manuf. spec.
Short term random uncertainty	180	409.64	47.2	Statistical process control [18]
Long term random uncertainty	172	392.16	43.3	Statistical process control [18]
Data acquisition	63	143.25	5.8	Agilent Data Acquisitions, manuf. spec
Dynamic pres. unc.	0	14	0.0	Est. based on unc. of 10 % *u*(*M*) and 5 % *u*(*γ*)
RSS	261	595.98	100	

**Table 7:** Stagnation temperature uncertainty for Stage 2 LP CFVs

Rosemont 162N100A	Relative uncertainty (*k* = 1)	Absolute uncertainty	Percent contribution	Comments
LP CFV stagnation temp., *T*_0_ = 294 K	(× 10^−6^)	(Pa)	(%)	
Thermistor transfer standard	4	1.2	0.0	Thermoetrics TS8901 (calibrated at NIST)
Uniformity of temperature bath	3	1	0.0	Expt. varied location of temp. std.
Fit residuals	3	10	2.0	Calibration data
Drift limit	68	20	7.8	Manuf. spec
Probe heat transfer effects	34	10	2.0	Expt. varied current
Spatial sampling error	204	60	70.6	Expt. varied depth of temp. transducer
Data acq. system	102	30	17.6	Manuf. pec
Stag. vs. measured.	0	0.1	0.0	Est. based on unc. of 10 % *u*(*M*) and 5 % *u*(*γ*)
RSS	243	71	100	

**Table 8:** Stage 2 MP CFV uncertainty

Uncertainty of stage 2 MP discharge coefficient	Relative uncertainty (*k* = 1)	Normalized sensitivity Coefficient	Percent contribution	Comments
HP CFV discharge coefficient, *C*_d_ = 0.9936	(× 10^−6^)	(-----)	(%)	
MP CFV, (*C*_d_ = 0.9929)	713	1.0	68.9	Stage 2 calibration (corr. effects included)
HP CFV stagnation pres., (*P*_0_ = 9120.52 kPa)	278	1.03	11.1	See explanation below
MP CFV stagnation pres., (*P*_0_ = 2280.13 kPa)	190	1.0	4.9	See Table 6
HP CFV stagnation temp., (*T*_0_ = 295.00 K)	242	0.64	3.2	See explanation below and Table 13
MP CFV stagnation temp., (*T*_0_ = 293.00 K)	244	0.5	2.0	See explanation below
HP CFV critical flow function, (C* = 0.7097)	244	1.0	8.1	See explanation
MP CFV critical flow function, (C* = 0.6914)	0*	1.0	0	Perfectly correlated betw. Stages 2 and 3
HP CFV diameter, (*d* = 2.54 cm)	0	2.0	0	Zero uncertainty during calibration stage
MP CFV diameter, (*d* = 2.54 cm)	17	2.0	0.2	Est. unc. for Δ*T*_12_ = 2 ± K
Line packing effect	108	1.0	1.6	Based on expected *T* and *P* drift during cal.
RSS	859		100	

**Table 9:** Uncertainty components of the TMS K-factor

*K*-factor uncertainty for natural gas	Relative uncertainty (*k* = 1)	Normalized sensitivity Coefficient	Percent contribution	Comments
*q* = 0.22 m^3^ MS/s (2 CFVs opened)	(× 10^−6^)	(-----)	(%)	
Discharge coefficient, (*C*_d_ = 0.9936)	830	−1.0	33.0	From Stage 3 unc. anal. (corr. effects incl.)
Critical low unction, (C* = 0.7386)	713	−1.0	24.4	see Table 10
Compressibility factor, (*Z*_TMS_ = 0.8143)	577	−1.0	16.0	AGA 8 Thermodynamic Database; rect. dist.
Total pressure uncertainty	297	1.0	4.2	See explanation below
Total temperature uncertainty	447	1.0	9.6	See explanation below
Total species composition unc.	467	1.0	10.5	See Table 11
Total molec. mass unc.	<1	1.0	0.0	See explanation below
Frequency, (*f* = 45.9254 Hz)	5	1.0	0.0	Manuf. spec.
Throat diameter, (*d* = 2.5396 cm)	69	−2.0	0.9	Est. unc. for Δ*T*_12_ = 10 ± 2 K
Univ. gas constant, *R*_u_ = 8314.472 J/kmol · K)	2	$-\sqrt{3/2}$	0.0	See Ref. [17]
Line packing effect	171	1.0	1.4	Estimated from *P* & *T* drift
RSS	1444		100	

**Table 10:** Stage 4 uncertainty components for the critical flow function

Critical flow function for the nozzle bank	Relative uncertainty (*k* = 1)	Percent contribution	Comments
NB critical flow function, C^*^ = 0.7386	(× 10^−6^)	(%)	
Viral effects on the boundary layer and sonic line	419	34.5	Est. using anal. models [19–21], rect. dist.,
Uncertainty of the thermodynamic database	577	65.5	Comparison betwn. [23] & [24], rect. dist.
RSS	713	100	

**Table 11:** Normal Gas Composition and Uncertainty at the 95 % Confidence Level

Gas Species	Amount of substance fraction (*x_k_*)	Relative uncertainty (*k* = 2) (%)
Methane	0.908	0.25
Ethane	0.02	05
Propane	0.02	0.7
IsoButane	0.009	1.5
Butan	0.009	1.5
IsoPentane	0.004	1.5
Pentane	0.004	1.5
Hexane	0.001	1
Heptane	0.001	1
Carbon Dioxide	0.003991	1
Nitrogen	0.014982	1
Helium	0.000997	1
Hydrogen	0.004	1

**Table 12:** Typical uncertainty of Rosemount 3051 used for Stage 4 pressure measurements

Rosemount 3051	Relative uncertainty (*k* = 1)	Absolute uncertainty	Percent contribution	Comments
TMS Press., *P* = 7175.08 kPa	(× 10^−6^)	(Pa)	(%)	
Calibration transfer standard (Ametek EPC 2000)	290	2080.5	69.7	Traceability to Ruska dead weight tester
Short term random uncertainty	160	1147.9	21.2	Statistical process control [18]
Long term random uncertainty	100	717.4	8.3	Statistical process control [18]
Data acquisition (Agilent 349070)	31	219.17	0.8	Manuf. spec.
RSS	347	2491.7	100	

**Table 13:** Typical Uncertainty for Temperature Measurements using Rosemount 3144 RTD

Rosemount 3144 RTD	Relative uncertainty (*k* = 1)	Absolute uncertainty	Percent contribution	Comments
HP CFV Temp., *T* = 285 K	(× 10^−6^)	(Pa)	(%)	
Temperature transfer standard	98	28.0	9.0	Manuf. spec (Hart Scientific 1521)
Digital accuracy	200	57.0	37.2	Manuf. spec (Rosemount 3144)
Drift	198	56.4	36.5	Manuf. spec (Rosemount 3144)
Ambient temperature effect	127	36.2	15.0	Manuf. spec (Rosemount 3144)
Data acquisition system	6	1.7	0.0	Manuf. spec (Agilent 349070 A)
Probe heat transfer effects	50	14.3	2.3	Stem conduction, radiation, & convection
RSS	328	96.7	100	

**Table 14:** Uncertainty budget for the volumetric flow of a MUT at the CEESI Iowa Natural Gas Calibration Facility

MUT volumetric flow unc.	Relative uncertainty (*k* = 1)	Normalized sensitivity Coefficient	Percent contribution	Comments
*q*_MUT_ = 1.3 m^3^/s (3 TMS in use)	(× 10^−6^)	(-----)	(%)	
*K*-factor, (*K* = 211.7 pulse/m^3^)	1373	1.0	86.9	From Stage 4 unc. anal. (corr. effects incl.)
Total pressure uncertainty	266	1.0	3.3	See explanation below
Total temperature uncertainty	376	1.0	6.5	See explanation below
Frequency, (*f* = 91.851 Hz)	5	1.0	0.0	See explanation below
Total species composition unc.	208	1.0	2.0	See explanation below
Total molec. mass unc.	0.0	1.0	0.0	Perfect correlation betw. *M_k_* at TMS & MUT
Total compressibility factor unc.	0.0	1.0	0.0	Perfect correlation betw. *Z*_MUT_ & *Z*_TMS_
Line packing effect	167	1.0	1.3	Estimated from *P* & *T* drift
RSS	1473		100	

## Footnotes

1 Although the OPR increases during flow diversion into or away from the collection tank, calculations indicate that it remains lower than the CPR.

2 For convenience, all uncertainty formulas give the square of the actual value unless otherwise noted.

3 Throughout this document certain commercial equipment, instruments, or materials are identified to foster understanding. Such identification does not imply recommendation or endorsement by the National Intstitute of Standards and Technology, nor does it imply that the materials or equipment identified are necessarily the best available for the purpose.

4 The short term and long term uncertainties discussed in later sections are also computed using this statistical procedure [18].
